# Supplementary material for: Conditional chemoconnectomics (cCCTomics) as a strategy for efficient and conditional targeting of chemical transmission
Source: eLife. 2024 Apr 30;12:RP91927. doi: 10.7554/eLife.91927 (PMC11060718; doi:10.7554/eLife.91927)
Supplement: Figure 3—source data 3. [file elife-91927-fig3-data3.zip › Figure 3 source data3 part 2.zip!/Figure 3-Source data 3-part2/1063-1-CRISPResso_Report_az97gqtx.zip!/CRISPResso_on_1063_4/9.Alleles_frequency_table_around_sgRNA_ggatgtttccggccaccgaa.pdf]

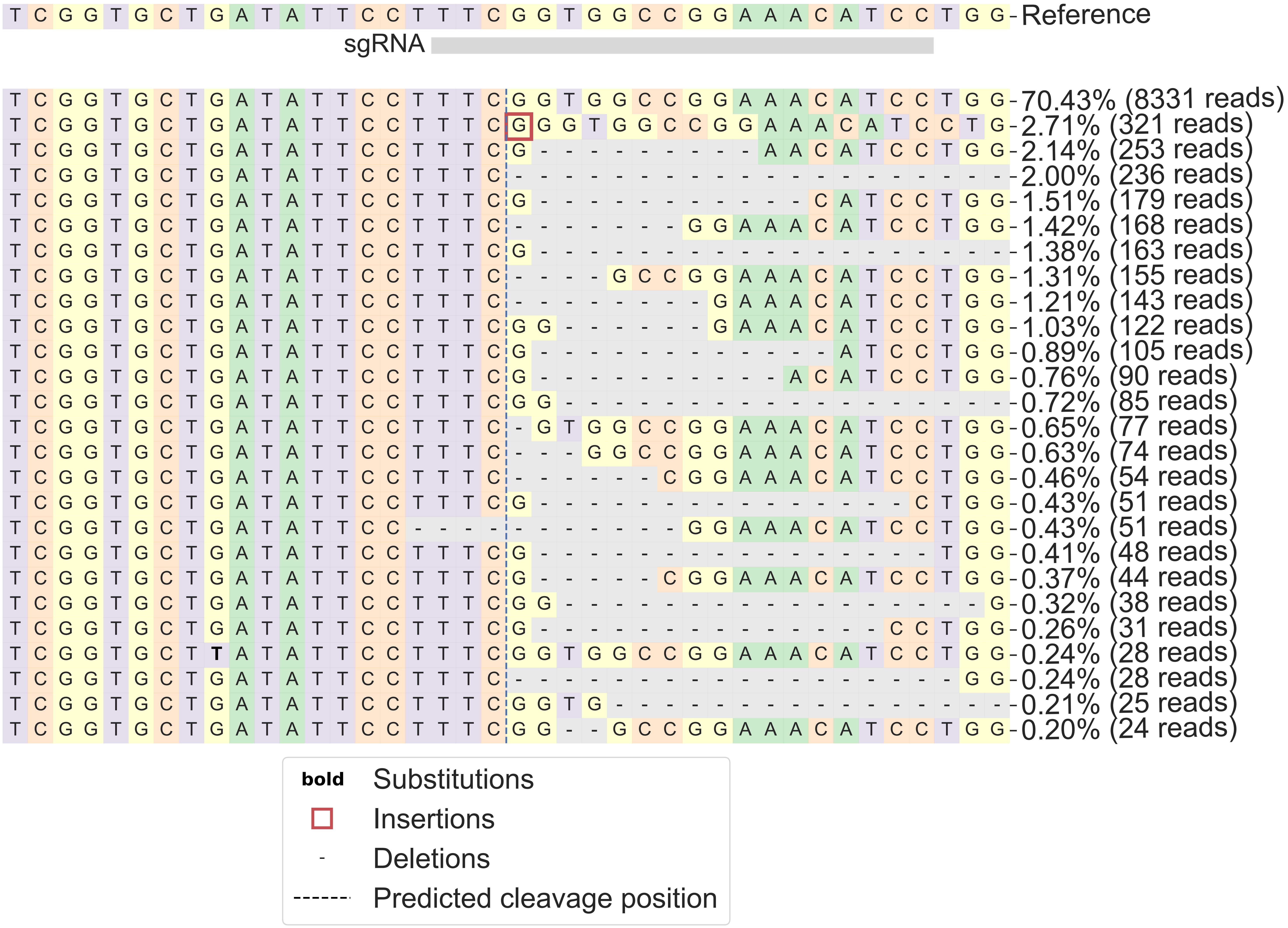

TCGGTGCTGATATTCCTTTCGGTGGCCGGAAACATCCTGG Reference
sgRNA
TCGGTGCTGATATTCCTTTCGGTGGCCGGAAACATCCTGG 70.43% (8331 reads)
TCGGTGCTGATATTCCTTTCGGGTGGCCGGAAACATCCTG 2.71% (321 reads)
TCGGTGCTGATATTCCTTTCG---------AACATCCTGG 2.14% (253 reads)
TCGGTGCTGATATTCCTTTC-------------------- 2.00% (236 reads)
TCGGTGCTGATATTCCTTTCG-----------CATCCTGG 1.51% (179 reads)
TCGGTGCTGATATTCCTTTC-------GGAAACATCCTGG 1.42% (168 reads)
TCGGTGCTGATATTCCTTTCG------------------- 1.38% (163 reads)
TCGGTGCTGATATTCCTTTC----GCCGGAAACATCCTGG 1.31% (155 reads)
TCGGTGCTGATATTCCTTTC--------GAAACATCCTGG 1.21% (143 reads)
TCGGTGCTGATATTCCTTTCGG------GAAACATCCTGG 1.03% (122 reads)
TCGGTGCTGATATTCCTTTCG------------ATCCTGG 0.89% (105 reads)
TCGGTGCTGATATTCCTTTCG----------ACATCCTGG 0.76% (90 reads)
TCGGTGCTGATATTCCTTTCGG------------------ 0.72% (85 reads)
TCGGTGCTGATATTCCTTTC-GTGGCCGGAAACATCCTGG 0.65% (77 reads)
TCGGTGCTGATATTCCTTTC---GGCCGGAAACATCCTGG 0.63% (74 reads)
TCGGTGCTGATATTCCTTTC------CGGAAACATCCTGG 0.46% (54 reads)
TCGGTGCTGATATTCCTTTCG---------------CTGG 0.43% (51 reads)
TCGGTGCTGATATTCC-----------GGAAACATCCTGG 0.43% (51 reads)
TCGGTGCTGATATTCCTTTCG----------------TGG 0.41% (48 reads)
TCGGTGCTGATATTCCTTTCG-----CGGAAACATCCTGG 0.37% (44 reads)
TCGGTGCTGATATTCCTTTCGG-----------------G 0.32% (38 reads)
TCGGTGCTGATATTCCTTTCG--------------CCTGG 0.26% (31 reads)
TCGGTGCTTATATTCCTTTCGGTGGCCGGAAACATCCTGG 0.24% (28 reads)
TCGGTGCTGATATTCCTTTC------------------GG 0.24% (28 reads)
TCGGTGCTGATATTCCTTTCGGTG---------------- 0.21% (25 reads)
TCGGTGCTGATATTCCTTTCGG--GCCGGAAACATCCTGG 0.20% (24 reads)
bold Substitutions
Insertions
- Deletions
Predicted cleavage position
